# Effect of Multivalent Cations on Intermolecular Association of Isotactic and Atactic Poly(Methacrylic Acid) Chains in Aqueous Solutions

**DOI:** 10.3390/polym11040605

**Published:** 2019-04-02

**Authors:** Patricija Hriberšek, Ksenija Kogej

**Affiliations:** Department of Chemistry and Biochemistry, Faculty of Chemistry and Chemical Technology, University of Ljubljana, SI-1000 Ljubljana, Slovenia; patricija.hribersek@fkkt.uni-lj.si

**Keywords:** polymethacrylic acid, stereoregularity, atactic, isotactic, multivalent cations, microgel-like particles, light scattering

## Abstract

The formation of nanoparticles of two poly(methacrylic acid) (PMA) isomers, atactic (aPMA) and isotactic (iPMA), was investigated in aqueous solutions in the presence of mono- (Na^+^) and multivalent cations (Mg^2+^ and La^3+^). Using dynamic (DLS) and static light scattering (SLS), we show that PMA nanoparticles have characteristics of microgel-like particles with a denser core and a swollen corona. iPMA aggregates are stable at a much higher degree of neutralization (*α*_N_) than the aPMA ones, indicating a much stronger association between iPMA chains. This is explained by proposing segregation of ionized and unionized carboxyl groups within the iPMA aggregates and subsequent cooperative hydrogen-bonding between COOH groups. The calculated shape parameter (*ρ*) suggests different behavior of both isomers in the presence of Mg^2+^ ions on one hand and Na^+^ and La^3+^ on the other. The microgel-like particles formed in the presence of Mg^2+^ ions have a more even mass distribution (possibly a no core-shell structure) in comparison with those in the presence of Na^+^ and La^3+^ ions. Differences between the aggregate structures in the presence of different ions are reflected also in calorimetric experiments and supported by pH and fluorimetric measurements. Reasons for different behavior in the presence of Mg^2+^ ions lie in specific properties of this cation, in particular in its strong hydration and preference towards monodentate binding to carboxylate groups.

## 1. Introduction

Interactions between macromolecules and metal ions play a very important role in biological processes in living systems, as well as in the technological and pharmaceutical fields [1,2,3]. Since approximately half of all proteins need a metal cofactor for stabilizing their structure, understanding of these interactions on the chemical and biological levels is a major challenge [1]. Not only electrostatic interactions [4] but also counterion dehydration [5,6] and entropically favored replacement of monovalent metal ions by multivalent ones [7] are the basis for association processes. Metals are indispensable for enzyme activity, in several catalytic reactions and for triggering cellular responses. Metalloproteins are responsible for the respiration function. For example, iron in the hemoglobin molecule reversibly binds oxygen. Metalloproteins that contain divalent cations like Mg^2+^, Ca^2+^ and Zn^2+^ are the most common and thus widely studied [1,8,9].

Mg^2+^ ions play a vital role in biochemical processes in the human body. They are the counterions to the ATP molecule [10], they stabilize nucleic acids by screening the repulsion between negatively charged phosphates [1] and they are also essential in stabilizing DNA geometry [1,11]. Mg^2+^ (besides Ca^2+^) stabilizes membranes by neutralizing charges in the bilayers because it binds to the negatively charged head groups of lipids [1,10]. Mg^2+^ is such an important cofactor in biochemistry because it displays some very characteristic features [10,12]. The most important among them are its small ionic radius and significantly larger hydrated radius, strong binding of hydration water (which makes it very hard to remove the hydration shell around the ion) and consequently slow exchange rate of water molecules in the hydration shell of the cation. Mg^2+^ ions form a hexa-coordinated complex with water and nature uses these water-rich Mg^2+^ complexes for providing enough water to the active sites of proteins [1,8,9,12]. Mg^2+^ is a so-called hard cation, which means that it prefers to interact with hard anions (such as the carboxyl groups), i.e., primarily with ligands that include oxygen as the coordinating atom [1]. Most protein structures coordinate Mg^2+^ with at least one carboxylate ligand. Mg^2+^ is bound to COO^−^ via only one oxygen atom, that is in so-called monodentate mode [1,8,9,13,14,15]. This binding mode is preferable due to the bulky hydration sphere of Mg^2+^ and consequently due to more favorable hydrogen-bonding interactions between the metal-free oxygen atom of COO^−^ and the neighboring water molecule, which are coordinated to Mg^2+^ (for easier visualization see Scheme S1 in Supporting Materials (SM)) [13]. Another possible binding mode with carboxylates is bidentate, which means binding of metal cation to both oxygen atoms of COO^−^. This is more common for Ca^2+^ binding to COO^−^ [9,13]. Another interesting property of Mg^2+^ is its ability to form so-called indirect or outer-sphere complexes with carboxylate ligand (via water molecules) apart from direct inner-sphere complexation directly with the COO^−^, in which the cation dehydrates. Only a few other metals have the potential to perform such interactions [1,12,16,17].

Trivalent cations, such as La^3+^, also have an important role in biological processes [18]. They can act as calcium analogs in living systems and may thus substitute Ca^2+^ in many proteins [19,20] and even in cell membranes [21] due to having a similar ionic radius (which is 1.0 Å for Ca^2+^ and 1.18 Å for La^3+^ [22]). In most cases, La^3+^ can replace Ca^2+^ bound to membranes and inhibit Ca^2+^ functions. However, trivalent cations are not transported across the cell membrane. Lanthanides can thus be used as probes for Ca^2+^ in biological systems [18,19,20,21] and as probes for interactions of metal ions with polyelectrolytes in solution in general when the role of counterion charge is investigated [23].

In this paper, we investigate the effect of multivalent cations on interchain association in aqueous solutions of isotactic (iPMA) and atactic poly(methacrylic acid) (aPMA). Association is ubiquitous in solutions of natural polymers and thus understanding of association processes is very important. Instead of natural polymers that often exhibit very complex behavior, simpler synthetic polyelectrolytes are used. Our motivation for studying PMA lies in the fact that it is the simplest polyelectrolyte with a cooperative conformational transition in aqueous solutions resembling those of biopolymers. The basis for cooperativity is usually intra- and/or intermolecular association. The studied multivalent metal cations are Mg^2+^ and La^3+^ and for comparison reasons the monovalent Na^+^ ion is included as well. Our main aim is to determine the characteristics of associates that form in the presence of Mg^2+^ and La^3+^ and compare these to those in the presence of Na^+^ [24,25,26,27]. Furthermore, we investigate how association affects the well-known conformational transition of the PMA chain upon ionization. For this purpose, we use dynamic (DLS) and static light scattering (SLS), pH, fluorescence and calorimetric measurements.

Several studies have recently been reported on the association behavior of aPMA and iPMA chains in aqueous solutions with added monovalent salts (LiCl, NaCl and CsCl) [24,25,26,27,28], whereas less is known on their interactions in systems with higher valent cations [29,30,31,32,33]. Only few contributions were published where the authors compared how effectively selected divalent cations bind to iPMA and syndiotactic PMA (sPMA) chains using potentiometric, equilibrium dialysis and viscometric techniques [30,31,32,33]. The results suggested that Mg^2+^ interacted more weakly with iPMA chains in comparison to Zn^2+^, Cu^2+^, Co^2+^ and Ni^2+^ [30,31,32,33], while it had a higher affinity for sPMA in comparison to Cu^2+^ [30,33]. Mg^2+^ binds more weakly also to carboxyl groups in poly(acrylic acid) (PAA) [34].

Luminescence studies of lanthanide complexes of all three PMA isomers (aPMA, sPMA and iPMA) [29] showed that three carboxylate groups bind to one trivalent cation in a bidentate fashion, which was also independently proposed by other groups [8,13,35,36]. In order to bind to carboxylate groups, the La^3+^ needs to lose some of the coordinated water molecules (the coordination number of La^3+^ is usually 9). This loss is detected as an increase in the cation’s luminescence intensity [29,36,37]. Results show that 3 carboxylate groups exchange 5–6 water molecules from the first coordination sphere of La^3+^ [29], which leads to an increase of entropy and a decrease of solvation free energy of the system [8]. In this way, trivalent cations bind to COO^−^ groups much more strongly than do the monovalent ones [29]. It is also known that the formation of lanthanide complexes with carboxylates takes place as inner-sphere complexation [29,38,39]. The aim of our study is to add to the understanding of these complex interactions and binding modes in solutions of synthetic polymers (polyelectrolytes) in the presence of multivalent ions, i.e., isotactic and atactic PMA.

## 2. Materials and Methods

### 2.1. Materials

The aPMA sample with the product number P2419-MAA (weight and number average molar masses are *M*_w_ = 159,900 g·mol^−1^ and *M*_n_ = 123,000 g·mol^−1^, polydispersity index is PDI = 1.3) was purchased from Polymer Source Inc (Montreal, QC, Canada). iPMA was obtained by hydrolysis of its ester form, the isotactic poly(methylmethacrylate) (iPMMA) which was synthesized according to the procedure reported in the literature at the Catholic University of Leuven [27,40]. The ester form (with 94% of isotactic, 4% of syndiotactic and 2% of atactic triads, *M*_w_ = 138,000 g·mol^−1^ and PDI = 4.6) was converted into the acid form (iPMA) first by acidic hydrolysis with concentrated sulfuric acid under the nitrogen flow followed by base hydrolysis adding sodium hydroxide. For final purification of the sample, the dialysis was used. First, the polymer was dialyzed against water to remove excessive NaOH and then against 0.02 M HCl to exchange Na^+^ for H^+^. For *α*_N_ < 0.2, iPMA precipitated from the solution. Further dialysis was performed against single and triple distilled water in order to thoroughly remove all low molar mass impurities. The obtained precipitate was filtered and dried by lyophilization (using Heto HETOSTATIC, Type CD 2.5; Heto-Holten A/S, Allerød, Denmark). The *M*_w_ and PDI of the purified iPMA (*M*_w_ = 69,500 g·mol^−1^ and PDI = 3) were determined by size exclusion chromatography and the degree of hydrolysis (>98%) was determined by ^1^H nuclear magnetic resonance measurements [27,41].

NaCl was purchased from Merck Milipore (>99%, Darmstadt, Germany), MgCl_2_ from Sigma Aldrich (>98%, St. Louis, Missouri, USA) and LaCl_3_ from Fluka in the form of LaCl_3_ × 7H_2_O (>65.5–70%, St. Louis, MO, USA). These salts were used to prepare stock solutions in water. The exact concentration of salts (*c*_s_) in stock solutions was determined by potentiometric titration using a standardized AgNO_3_ solution. Pyrene (optical grade) for fluorimetric measurements was purchased from Aldrich (Darmstadt, Germany). The saturated solutions of pyrene in NaCl, MgCl_2_, and LaCl_3_ stock solutions were prepared as reported previously [42,43].

### 2.2. Preparation of Solutions

aPMA is soluble in water at all *α*_N_, while iPMA is insoluble at *α*_N_ < 0.2; therefore, two different procedures were used to prepare the PMA solutions.

Stock solution of aPMA was prepared in water at *α*_N_ = 0 by dissolving dry polymer in water. After one day of stirring, the solution was filtered through 0.45 μm Millex HV filter and the polymer concentration (*c*_m_, in g·L^−1^) was determined by potentiometric titration with a standardized NaOH solution of known concentration. Solutions of aPMA with a desired salt concentration were prepared by adding calculated volumes of concentrated salt solutions to aPMA stock solution in water to adjust the ionic strength (*I*) to 0.01 mol·L^−1^. The concentration of aPMA in final solutions used for calorimetric measurements was *c*_m_ = 1 g·L^−1^ at *α*_N_ = 0 (corresponding to the molar concentration *c*_p_ = 0.012 mol·L^−1^, expressed in moles of COOH groups per volume).

Starting solution of iPMA was first prepared at a higher *α*_N_ value (*α*_N_ > 0.5, well above the solubility limit) in order to assure that the polymer was completely dissolved. This was done by a stepwise addition of a calculated volume of 1 M NaOH to a suspension of polymer in water. At the start, the solution was heated to around 50 °C for one hour and then stirred for several days at room temperature. When the polymer was visually dissolved, the solution was filtered through 0.45 μm Millex HV filter. The precise values of α_N_ and *c*_m_ were determined after filtration by potentiometric titration first with a standardized 0.1 M NaOH solution in the direction of increasing α_N_ and then with a standardized 0.1 M HCl solution in the direction of decreasing *α*_N_. Lower *α*_N_ values were obtained by a stepwise addition of a calculated amount of 0.1 M HCl solution. To adjust the *c*_m_ and *I* to the desired final values, samples were diluted with triple distilled water. A calculated amount of the concentrated salt solution was added to this starting solution to achieve the desired *I*. The *I* value was calculated from *I* = 1/2∑_i_*c*_i_*z*_i_^2^, where sum is taken over ionic species (i) of the added electrolyte with molar concentration *c*_i_ and charge number *z*_i_, and was set to *I* = 0.01 mol·L^−1^ for all chlorides. The concentration of iPMA in final solutions was the same as that of aPMA (i.e., *c*_p_ = 0.012 mol·L^−1^).

Due to too weak scattering in PMA solutions with *c*_m_ = 1 g·L^−1^ (used in calorimetry), *c*_m_ for LS measurements was set to a twice higher value: *c*_m_ = 2 g·L^−1^ at *α*_N_ = 0 or *c*_p_ = 0.023 mol·L^−1^. With aPMA (*α*_N_ = 0), it was also necessary to increase *I* to a 10-times higher value (*I* = 0.1 mol·L^−1^) for all added salts. Additionally, measurements with aPMA were also performed at *I* = 0.2 and 0.05 mol·L^−1^ for NaCl and LaCl_3_, respectively, in order to see the effect of ionic strength on LS results. All *I* values employed in LS measurements for aPMA are reported in Table 1. For convenience, the molar concentration (*c*_s_) of the metal chlorides is given as well. The same *c*_m_ and *I* conditions were then used also for pH and fluorimetric measurements.

The starting iPMA solution had *α*_N_ > 0.5 (see above) and the target in the final solution was *α*_N_ = 0.2. We tried to prepare iPMA solutions with *α*_N_ ≈ 0.2 and *c*_p_ = 0.022 mol·L^−1^ (corresponding to *c*_m_ = 2 g·L^−1^ at *α*_N_ = 0) at the same *I* (= 0.1 mol·L^−1^) as for aPMA. This was possible only in the presence of NaCl, where no precipitation occurred when *α*_N_ was reduced to *α*_N_ ~ 0.2. However, in MgCl_2_ and LaCl_3_ solutions iPMA (*α*_N_ > 0.5) started to precipitate if *I* was too high (*I* = 0.1 mol·L^−1^). Consequently, all iPMA solutions were prepared at a lower *I* than solutions of aPMA so that precipitation was avoided. Thus, *I* was 0.01 mol·L^−1^ in NaCl (*c*_s_ = 0.01 M) and MgCl_2_ (*c*_s_ = 0.0033 M; both with *α*_N_ = 0.19) and it was even lower in LaCl_3_ (*I* = 0.005 mol·L^−1^, corresponding to *c*_s_ = 0.00083 M LaCl_3_; *α*_N_ = 0.22). All *I*, *c*_s_, and *α*_N_ values for iPMA are reported in Table 1.

After three days of stirring, the iPMA samples were filtered immediately through 0.22 μm Millex HV filters directly into LS cells. The aPMA samples were left to rest before filtration due to the tendency of aPMA to form associates under shear (imposed by mixing) [24,44,45] and were filtered one day later. Both PMA solutions were left standing at room temperature for approximately 3 days before the LS measurements were performed.

The same samples as used for LS were also employed to measure the pH of solutions, whereas for fluorimetry solutions were prepared in pyrene saturated solvents as reported previously [42,43].

### 2.3. Methods

*Calorimetry.* Isothermal Titration Calorimetry (ITC) was used to determine the ionization enthalpies of both PMA isomers. ITC measurements were carried out in the TAM 2277 calorimeter (Thermometric AB, Sweden). The ionization enthalpies (Δ*H*_ion_) for both PMAs were measured as protonation enthalpies (Δ*H*_prot_), i.e., for the opposite reaction. The calorimeter cell was filled with 2 mL of PMA with *c*_p_ = 0.012 g·mol^−1^ previously neutralized to the desirable initial *α*_N_ value in order to completely dissolve the polymer (iPMA). In the calorimeter, both aPMA and iPMA solutions were titrated back to *α*_N_ = 0 with 0.06 M HCl solution. The titrant (HCl solution) was added into the cell in small volume increments (usually 5 μL) with a motor run syringe up to the total volume of 250 μL. Care was taken that all solutions were degassed prior to the measurements. The calorimeter was calibrated by passing a known electric current through an electric heater with a known electric power. Due to a broad range of *α*_N_ values that had to be covered in certain cases, some data were collected in two runs, starting from different initial *α*_N_ values. The measured heat effects were corrected for the enthalpies of dilution of PMA and HCl in the appropriate solvent. These heats were measured in a separate run and were found to be negligible in comparison to Δ*H*_prot_.

*Light scattering*. DLS and SLS measurements were performed with the 3D-cross-correlation spectrometer from LS Instruments GmbH (Fribourg, Switzerland) [46]. The source of incident light was the He-Ne laser with a wavelength *λ*_0_ = 632.8 nm. Intensity of scattered light was collected in the angular range between 40° and 150° with a step of 10° after thermostating the samples at 25 °C for 30 min. At each angle, five correlation functions were collected and averaged. For processing the measured correlation functions, CONTIN analysis was used. Examples of the calculated distributions of hydrodynamic radii (*R*_h_) are shown in Figure S1 in SM for selected solutions of iPMA and aPMA. Hydrodynamic radii of large particles were obtained by extrapolation of the *R*_h_ values measured at different angles to *θ* = 0°. Radii of gyration (*R*_g_) were determined from the angular dependency of the LS intensity. It was ascertained through the analysis of the LS data that large particles correspond to associates of several chains (see below), therefore designations *R*_h,ass_ and *R*_g,ass_ are used for their hydrodynamic radii and radii of gyration, respectively. From *R*_h,ass_ and *R*_g,ass_, the shape parameter (*ρ*) was calculated as *ρ* = *R*_g,ass_/*R*_h,ass_. *R*_h_ of individual chains and eventual other smaller particles (*R*_h,1_ and *R*_h,2_) did not depend on angle and are therefore reported as average values of *R*_h_ measured at several angles (see Tables S1 and S2, SM). All details of LS data evaluation were reported previously [24,25]. Examples of data analysis for selected samples in this study are presented in SM (Figures S1–S3).

*pH measurements*. The pH values of samples were determined using the combined glass micro-electrode from Mettler Toledo (Schwerzenbach, Switzerland) type InLab^®^ 423 and the Iskra pH meter model MA 5740 (Ljubljana, Slovenia).

*Fluorimetry*. Pyrene was used as the external fluorescence probe to analyze micropolarity in iPMA and aPMA solutions. The preparation of water or aqueous salt solutions saturated with pyrene was as reported previously [42,43]. These solutions were then used as solvents for the preparation of PMA solutions. The fluorescence emission spectra of pyrene were recorded on a Perkin-Elmer model LS-100 luminescence spectrometer at 25 °C. The excitation light wavelength was 330 nm and the emission spectra were collected in the wavelength region 350–450 nm. The recording speed was 100 nm·min^−1^. Five emission spectra were collected for each sample and averaged. From the averaged spectra, the ratio of intensities of the first (*I*_1_) and the third (*I*_3_) vibrational peak in the emission spectrum of pyrene (*I*_1_/*I*_3_) was calculated.

## 3. Results

### 3.1. Calorimetry

**Figure 1:**
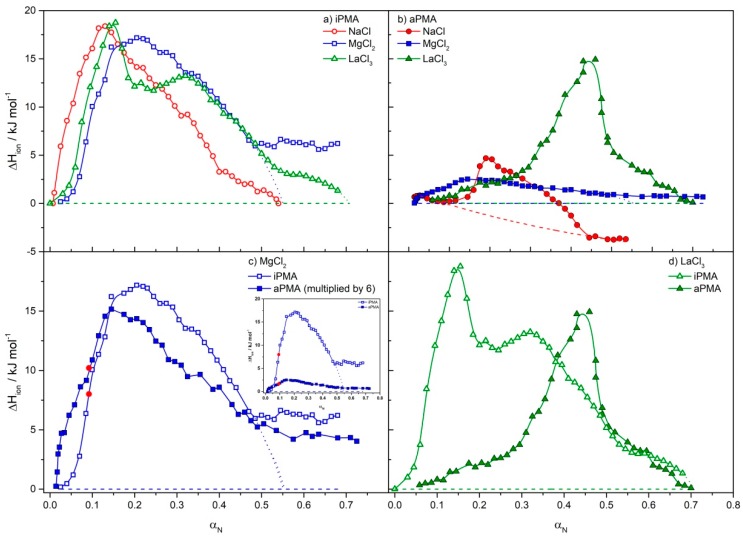
Ionization enthalpies (Δ*H*_ion_) in dependence on *α*_N_ for (**a**) iPMA and (**b**) aPMA (*c*_m_ = 1 g·L^−1^) in aqueous solutions of NaCl, MgCl_2_ and LaCl_3_ with *I* = 0.01 mol·L^−1^ at 25 °C; (**c**) comparison of calorimetric curves for iPMA and aPMA in 0.00333 M MgCl_2_; the Δ*H*_ion_ values for aPMA are inhere multiplied by 6 (the inset in Figure 1c shows comparison of the original Δ*H*_ion_ values). The red circles in these curves indicate *α*_N_ at which the solution became opaque in the direction of decreasing *α*_N_; (**d**) comparison of calorimetric curves for iPMA and aPMA in 0.00167 M LaCl_3_. The solid lines are best fits of the experimental data and are used merely to lead the eye. The red, blue and green dashed lines in (**a**–**d**) are the base lines used for the integration of the peaks (see text).

It is known [26,47] that the conformational transition of the PMA chain is reflected in the ITC (Δ*H*_ion_ versus α_N_) curves as an endothermic peak. Precise ITC measurements in aqueous PMA solutions with added 0.01 M alkali chlorides (XCl) [26] revealed that the change in chain conformation is the main process that contributes to Δ*H*_ion_ in the aPMA case. On the other hand, an additional process was clearly identified for iPMA [26], which was assigned to strong intermolecular association and contributed to around 50% of the total transitional enthalpy of iPMA in 0.01 M XCl solutions (X = monovalent cation). Similar ITC thermograms with well-pronounced endothermic peak(s) were obtained in this study for both PMAs in aqueous solutions with added metal chloride salts with different cation valency (Na^+^, Mg^2+^ and La^3+^; see Figure 1a,b for iPMA and aPMA, respectively). The ionic strength of all chloride solutions in these measurements was *I* = 0.01 M, the same as in reference [26].

The integration of the area under the peak in the Δ*H*_ion_ vs. α_N_ curves gives the enthalpy change associated with the conformational transition (Δ*H*_conf_) and eventual other processes taking place in solution upon ionization/protonation of carboxyl groups on the PMA chain (see Δ*H* values reported in Table 2). This calculation requires a base line. For the construction of the base lines, we have followed the same procedure as reported previously [26]. Previous data for iPMA obtained in NaCl solutions at different temperatures [26] showed that Δ*H*_ion_ values were approximately zero outside the region of the conformational transition. That outcome was anticipated in this case as well and therefore the drawn base lines in most cases fit the ordinate (see the dashed lines in Figure 1). An exception is the aPMA solution in NaCl, where the Δ*H*_ion_ values clearly drop below zero for *α*_N_ > 0.35 (after the superimposed endothermic peak). The base line in this case was constructed by comparing this plot with the ionization enthalpies of PAA [41,47], a polyelectrolyte without the conformational transition. In the PAA case, the ionization enthalpies are exothermic (without the superimposed endothermic peak) and gradually decrease with increasing *α*_N_, so as proposed by the base line drawn for aPMA in NaCl in Figure 1b. The uncertainty in the evaluation of Δ*H*_tr_ values was estimated to be around 10%.

For aPMA, the conformational enthalpy (Δ*H*_conf_) in 0.01 M NaCl determined in this study is Δ*H*_conf_ = 1.12 kJ·mol^−1^. This value is somewhat larger than the previously reported ones (Δ*H*_conf_ = 1.03 kJ·mol^−1^ in water [47] and Δ*H*_conf_ = 1.0 kJ·mol^−1^ in 0.01 M NaCl [26]). Taking into account that PMA samples in those studies had different molar mass and tacticity values, these differences are within the uncertainty limits of ITC measurements (±10%).

The focus in this study is on multivalent cations. The curve for aPMA in MgCl_2_ solution at *I* = 0.01 mol·L^−1^ (*c*_s_ = 0.0033 M) is distinctly different from the one recorded in 0.01 M NaCl. The measured heat effects for aPMA in MgCl_2_ are lower, besides the endothermic peak is rather broad and shifted to higher *α*_N_; it extends over a wide *α*_N_ region, from *α*_N_ ≈ 0 to *α*_N_ ≈ 0.55 as anticipated from the dotted blue line in Figure 1c. For comparison, the peak in NaCl solutions extends from *α*_N_ ≈ 0.15 to *α*_N_ ≈ 0.45. Moreover, heat effects in MgCl_2_ do not drop to zero for *α*_N_ > 0.55, but reach some kind of a plateau with constant Δ*H*_ion_ (≈ +0.73 kJ·mol^−1^) values (for more details see Figure 1c, where Δ*H*_ion_ values in aPMA solution in the presence of MgCl_2_ are multiplied by a factor of 6 for clearer comparison of the shape of this curve with that for iPMA). Obviously, curve shapes for aPMA and iPMA are similar, but Δ*H*_ion_ values are higher for iPMA (see discussion below). Visual observation of aPMA solution in the presence of Mg^2+^ ions shows that the solution turns weakly opaque at α_N_ ≈ 0.1 (this point is indicated by the red circle in the enlarged plot in Figure 1c) and becomes completely transparent again when *α*_N_ approaches 1. However, no precipitation is observed over the entire α_N_ region scanned in the calorimetric experiment. In the integration of Δ*H*_ion_ curves in the presence of MgCl_2_ (for both PMAs), this plateau was omitted and only the broad peak area was integrated, as suggested by the dotted blue line (see also arguments in Discussion).

Based on these findings, and in agreement with features of calorimetric curves for iPMA, the heat obtained by integration of the broad peak in 0.0033 M MgCl_2_ is attributed to two events that are probably taking place simultaneously in this case: (i) to the change in chain conformation, accompanied by Δ*H*_conf_ (similar to 0.01 M NaCl), and (ii) to intermolecular association, which is induced by the presence of divalent Mg^2+^ ions and accompanied by so-called association enthalpy (Δ*H*_ass_). The presumption on association was confirmed by LS, which identified large particles in solutions (see below). Therefore, a more general term transition enthalpy (Δ*H*_tr_) is used in this case for the total heat effect, which includes both, the change in conformation and intermolecular association: Δ*H*_tr_ = Δ*H*_conf_ + Δ*H*_ass_. It is equal to Δ*H*_tr_ ≈ 0.83 kJ·mol^−1^ for aPMA in aqueous 0.0033 M MgCl_2_ at 25 °C (see values reported in Table 2).

It can be seen from Figure 1b and the data in Table 2 that heat effects (Δ*H*_ion_) for aPMA in 0.00167 M LaCl_3_ are between 2.5- and 3.5-times higher than in 0.01 M NaCl and 0.0033 M MgCl_2_, respectively, and the endothermic peak is shifted to higher *α*_N_; it extends from *α*_N_ ≈ 0.1 to *α*_N_ ≈ 0.7. The solution of aPMA in LaCl_3_ turned opaque at a similar *α*_N_ value as in the case of MgCl_2_, which again indicates that a more extensive association of aPMA chains is occurring. The shape of the Δ*H*_ion_ curve suggests that the nature of association/aggregation and conformational transition in the presence of trivalent La^3+^ cations is different from that in the presence of divalent Mg^2+^ cations, as the peak apex is shifted to considerably higher *α*_N_ (centered at *α*_N_ close to 0.5) and is accompanied by a shoulder at low *α*_N_.

Results of calorimetric measurements for iPMA in 0.01 M NaCl, 0.0033 M MgCl_2_ and 0.00167 M LaCl_3_ solutions are presented in Figure 1a and the calculated Δ*H*_tr_ values are reported in Table 2. A more straightforward comparison of the curves for both PMAs in the presence of multivalent chlorides is shown in the inset of Figure 1c (for 0.0033 M MgCl_2_) and in Figure 1d (for 0.00167 M LaCl_3_). The shape of the Δ*H*_ion_ vs. *α*_N_ curve for iPMA in 0.0033 M MgCl_2_ is very similar to the one for aPMA, only Δ*H*_ion_ values for aPMA are lower, which can be very clearly seen in Figure 1c and Table 2. In Figure 1c, the Δ*H*_ion_ values for aPMA are multiplied by a factor of 6 to highlight a similar shape of the curve as in the iPMA case (the inset in Figure 1c shows comparison of the original Δ*H*_ion_ values). The broad peak in iPMA solution extends up to *α*_N_ ≈ 0.55, just as in the aPMA case, and the Δ*H*_ion_ values do not drop to zero for *α*_N_ > 0.55, but remain constant at around 5 kJ·mol^−1^ (these plateau values are lower in the aPMA case, i.e., around 0.73 kJ·mol^−1^). The highlighted shape suggests a similar mode of interactions in the presence of divalent Mg^2+^ cations for both isomers, which is related to the specific features of Mg^2+^ cations (see Discussion). The integration of calorimetric curves results in considerably (two to almost seven times) higher Δ*H*_tr_ values for iPMA in the presence of all three chlorides, in particular in MgCl_2_ solutions (c.f. Table 2). Based on these features, we suggest that iPMA chains are strongly associated in the presence of all three metal chlorides, while intermolecular association in the aPMA case is weaker (MgCl_2_ and LaCl_3_) or mostly absent (NaCl).

One very distinct feature of the Δ*H*_ion_ curve for iPMA in 0.00167 M LaCl_3_ is that it displays two well resolved peaks, while in the aPMA case the first peak (at low *α*_N_) is much lower and therefore less expressed (see Figure 1d). This suggests that the proposed processes, conformational transition and association, are well separated in both PMA solutions in the presence of 0.00167 M LaCl_3_. Following the curve for iPMA/LaCl_3_ in the direction of decreasing *α*_N_, first the change in conformation takes place (c.f. the lower and broader peak), followed by inter-chain association at sufficiently low *α*_N_ (≤0.3; c.f. the higher peak), which eventually leads to precipitation of the weakly charged and strongly aggregated polymer from the solution. The latter peak is much less pronounced in aPMA/LaCl_3_ solutions. The evaluated Δ*H*_tr_ term in the LaCl_3_ solutions thus includes the conformational term (Δ*H*_conf_) and the association and precipitation terms, which cannot be separated and are both included in Δ*H*_ass_. The intermolecular association (and precipitation) in aPMA/LaCl_3_ solution evidently contribute less to Δ*H*_tr_ in comparison to iPMA/LaCl_3_ solution.

In view of conditions for LS measurements, calorimetric measurements were performed also at a 10-times higher metal chloride concentrations for some selected cases (i.e., for 0.0333 M MgCl_2_ and 0.0167 M LaCl_3_, which corresponds to *I* = 0.1 mol·L^−1^). At higher *I* the electrostatic screening is more efficient and consequently intermolecular association between PMA chains is expected to be even more pronounced than at *I* = 0.01 mol·L^−1^. Both polymers precipitated from MgCl_2_ and LaCl_3_ solutions at *I* = 0.1 mol·L^−1^ and high *α*_N_. For aPMA, this happened at *α*_N_ ≈ 0.4 in MgCl_2_ and at *α*_N_ ≈ 0.5 in LaCl_3_ solution, whereas for iPMA at an even lower *α*_N_ (≈0.1), no heat effects could be measured above these points. The Δ*H*_ion_ vs. *α*_N_ curves for these cases are presented in Figure S4 (SM). By comparing the data in Figure 1 and Figure S4 we see that heat effects are significantly higher at higher *I*. Besides, the calorimetric curves do not display any significant peak, but instead Δ*H*_ion_ values are more or less constant (aPMA in MgCl_2_ and iPMA in LaCl_3_) or show a gradually increasing trend (aPMA in LaCl_3_). This is attributed to very extensive intermolecular association, which overruns heat effects due to the change in chain conformation or possibly even prevents the latter. Further analysis of these measurements was therefore not undertaken.

### 3.2. Light Scattering

It is appropriate to stress at the beginning that LS measurements could not be performed for both polyacids at the same *α*_N_ (for example either at *α*_N_ ≈ 0 or at *α*_N_ ≈ 0.2) because the aPMA aggregates disintegrate rapidly for *α*_N_ > 0, as demonstrated previously [25], and iPMA is not soluble at *α*_N_ ≈ 0 (see also Materials and Methods).

LS experiments identified two (in some cases three) populations of particles in iPMA and aPMA solutions (see examples of *R*_h_ distributions in Figure S1, SM). The *R*_h_ values of large particles (peak 2 in Figure S1a and peak 3 in Figure S1b) are considerably higher than *R*_h,1_ and *R*_h,2_ (peak 1 in Figure S1a and peaks 1 and 2 in Figure S1b) and are therefore assigned to interchain associates and designated as *R*_h,ass_. Associates are large enough to determine their radius of gyration (*R*_g,ass_) as well and therefrom the *ρ* values were calculated. All these LS parameters are reported in Table 3.

The data show that *R*_h,ass_ for iPMA associates in NaCl (59 and 80 nm,) and in MgCl_2_ (75 nm) are significantly smaller than those in LaCl_3_ (191 nm). The range of *R*_h,ass_ (172–200 nm) and *R*_g,ass_ (126–164 nm) values for aPMA associates is not so wide and aggregate size does not depend significantly on the type of the counterion. This may be attributed to the fact that aPMA has *α*_N_ = 0, whereas iPMA has a higher *α*_N_ (≈0.2) and the screening role of the added ions becomes important. The calculated *ρ* values in the presence of Na^+^ and La^3+^ ions are *ρ* = 0.69–0.71 for iPMA and somewhat higher, *ρ* = 0.73–0.77, for aPMA. Note that these *ρ* values are below the value for a hard sphere (*ρ* = 0.778) and were used in this study to evaluate the internal structure of the aggregates (see Discussion section). In both cases, *ρ* in the presence of MgCl_2_ is higher than that, at around 0.95.

### 3.3. pH Measurements

The pH values determined in PMA solutions in the presence of NaCl, MgCl_2_ and LaCl_3_ are reported in Table S3. They were measured in a broad range of *I* values. From the measured pH, the degree of ionization (*α*_i_) was calculated using the relationship *α*_i_ = *α*_N_ + ([H^+^] − [OH^−^])/*c*_p_ (here, [H^+^] and [OH^−^] are molar concentrations of hydronium and hydroxide ions), which follows from the electroneutrality condition.

**Figure 2:**
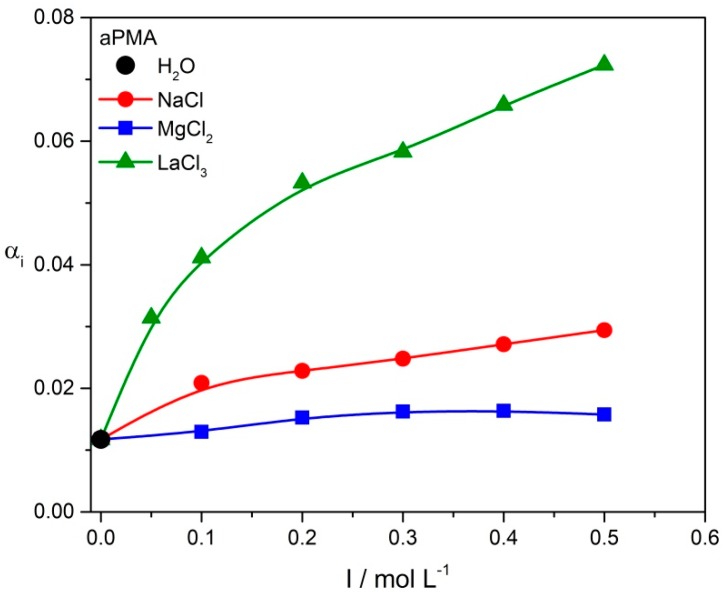
Calculated *α*_i_ values for aPMA (*c*_m_ = 2 g·L^−1^, *α*_N_ = 0) in dependence on *I* in aqueous NaCl, MgCl_2_ and LaCl_3_ solutions.

The *α*_i_ values reported in Table S3 show that added salts have a pronounced effect on *α*_i_ in aPMA solutions with *α*_N_ = 0. The calculated *α*_i_ values for aPMA are plotted in Figure 2 in dependence on *I* and clearly show the increase of *α*_i_ with increasing *I*. Around 1% (the calculated *α*_i_ is 0.012) of COOH groups on aPMA are ionized at *α*_N_ = 0 in water when no salt is added to the solution, whereas the addition of salt increases *α*_i_ by a factor of 2.5 in NaCl solutions (at the highest NaCl concentration) and even more significantly in LaCl_3_ solutions (by a factor of 6 at the highest LaCl_3_ concentration). This increase is attributed to the replacement of protons (H^+^) from the polymer’s COOH groups. A larger effect of La^3+^ ions in comparison with the Na^+^ ones is expected due to the fact that one La^3+^ is able to exchange three H^+^ ions. Unlike Na^+^ and La^3+^, no significant effect of the divalent Mg^2+^ ions on *α*_i_ values is detected: *α*_i_ increases from 0.012 (in water) to 0.016 in 0.5 M MgCl_2_.

The increase in *I* has no effect on *α*_i_ also in iPMA solutions with *α*_N_ ≈ 0.2, which applies to all added metal chlorides (*α*_i_ stays the same as *α*_N_; c.f. Table S3). Consequently, the *α*_i_ data for iPMA are not plotted in Figure 2. This result is expected, because iPMA is already significantly charged at *α*_N_ ≈ 0.2 and eventual replacement of a few H^+^ ions by metal cations should not affect the ionization degree of carboxyl groups significantly, as also demonstrated previously [48].

### 3.4. Fluorimetry

To detect the micropolarity of the aggregates’ interior of PMA isomers, fluorimetric measurements were performed. PMA does not have any fluorophore, therefore pyrene as an external fluorescence probe was used to monitor the formation of eventual hydrophobic domains [42,43,48] by following the pyrene fluorescence ratio *I*_1_/*I*_3_. The *I*_1_/*I*_3_ values are reported in Table 4. It can be seen that all *I*_1_/*I*_3_ values are below 1 for aPMA associates in the presence of all cations, which means that pyrene is located in a rather nonpolar environment, presumably offered by the interior of the aPMA associates. In contrast, the *I*_1_/*I*_3_ values are around 1.5 for iPMA solutions in NaCl and MgCl_2_, which indicates a much higher polarity of the aggregates’ interior in this case. For iPMA associates in aqueous LaCl_3_ at *I* = 0.005 mol·L^−1^ the *I*_1_/*I*_3_ ratio is around 1, similar to the aPMA case.

## 4. Discussion

The basis for understanding the behavior of both PMAs in water is the state of chains in solution at low *α*_N_. We therefore start the discussion with light scattering results obtained at low *α*_N_. In agreement with what was proposed in the presentation of calorimetric results above, the LS measurements reveal that large associates/aggregates are present in solutions when chains are not ionized (aPMA: *α*_N_ = 0) or just sufficiently ionized to get the polymer dissolved (iPMA: *α*_N_ ≈ 0.2; note that aggregates in aPMA solutions are no longer present at *α*_N_ above 0). The size of these aggregates is in the nanometer range (for example *R*_h,ass_ is 60–200 nm; c.f. Table 3).

**Figure 3:**
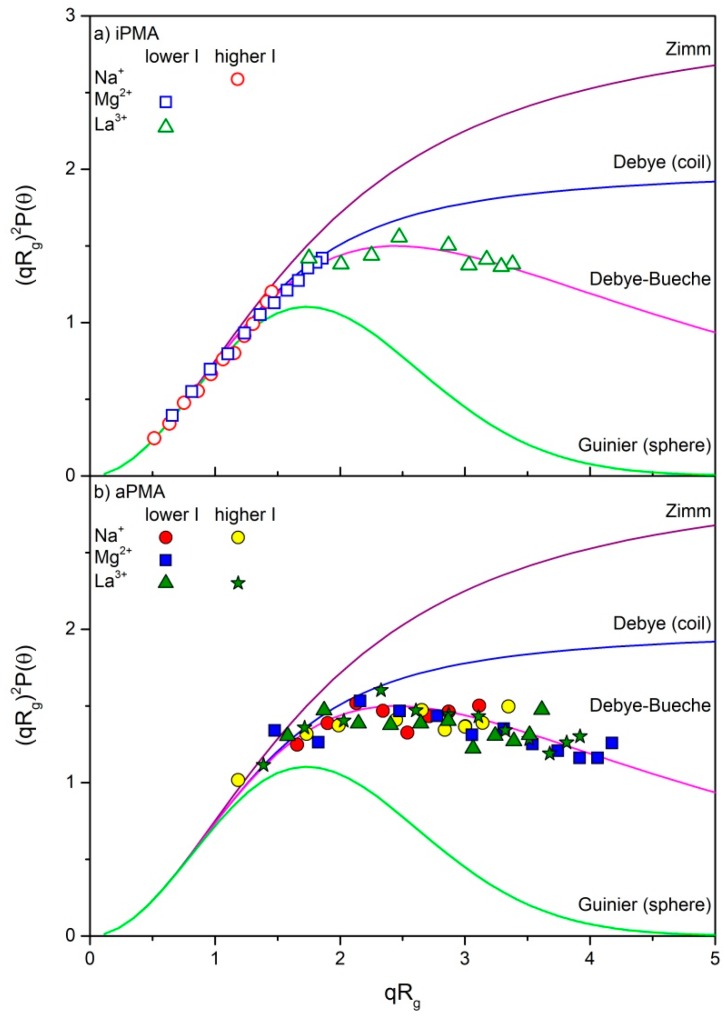
Kratky plot (the dependence of (*qR*_g_)^2^*P*(*θ*) on *qR*_g_) for four selected topologies (solid lines; for details on these scattering functions see [49]) and the experimental data for associates of (**a**) iPMA (different *α*_N_, see Table 1) and (**b**) aPMA (*α*_N_ = 0) in aqueous solutions of NaCl, MgCl_2_ and LaCl_3_ (for values of *I* see Table 1): *c*_m_ = 2 g·L^−1^.

To disclose the nature of the aggregates, the LS data are shown in Figure 3 in the form of a Kratky plot (i.e., the dependence of (*qR*_g_)^2^*P*(*θ*) on *qR*_g_, where *q* = (4π*n*_o_/*λ*_o_)sin(*θ*/2) is the scattering vector and *P*(*θ*) is so called particle scattering factor) and compared with calculated curves for some relevant topologies. Differences in topologies of particles can be reliably discussed for *qR*_g_ ≥ 2. The particles in aPMA solutions (*α*_N_ = 0) are large enough that the experimental points extend to *qR*_g_ close to 4, whereas in iPMA solutions (non-zero *α*_N_) they exceed this value only in the presence of LaCl_3_. However, in 0.0033 M MgCl_2_ the data approach *qR*_g_ ≈ 2, which offers the possibility to speculate about the nature of particles also in the iPMA case.

Data points in aPMA solutions (Figure 3b) obviously agree with the Debye-Bueche scattering function, which was derived for structures with a compact core, impermeable for the solvent, and a swollen outer corona that allows the solvent to penetrate through (for equation of this scattering function see SM). This is a typical structure of a microgel-like particle (see Scheme S2 in SM) [25]. At higher *qR*_g_ values (in particular in the presence of MgCl_2_ and LaCl_3_), the experimental points show an upturn from the Debye-Bueche towards the Debye scattering function. The latter function is valid for random coils with a more even mass distribution compared to microgel-like particles. This kind of positive upturns has already been observed in the literature [50,51,52] and was assigned to two possible effects: to excluded-volume interactions and/or chain stiffness. By taking into account that Mg^2+^ and La^3+^ ions, which facilitate association, are strongly hydrated (see the discussion below), the excluded-volume interactions are proposed to be operative in our case.

Similar to aPMA, the data points for iPMA in LaCl_3_ solution follow the Debye-Bueche function (Figure 3a) and suggest comparable aggregate topology as far as mass distribution is concerned. By taking into account the non-zero *α*_N_ with iPMA, intermolecular association seems to be much stronger in this case. One would namely expect a lower tendency towards association (or no association) at a higher polyion charge, as with aPMA.

The data for iPMA in the presence of 0.0033 M MgCl_2_ seem to follow the Debye function, but are limited to *qR*_g_ ≤ 2, therefore reliable conclusions are difficult. However, by also considering the higher *ρ* values obtained for both PMAs with added MgCl_2_ (*ρ* = 0.97 and 0.92 for iPMA and aPMA; respectively), this altogether suggests that particles are lacking the compact core in this case; they are more permeable for the solvent throughout and thus more coil-like. This will be clarified below by taking into account the monodentate binding mode to carboxylate groups known for Mg^2+^ ions.

Although LS identifies similar aggregates in iPMA and aPMA solutions as far as mass distribution is concerned, fluorescence spectroscopy reveals very different internal, and consequently also surface, polarity of these aggregates, which may only be caused by different chemical composition, i.e., different distribution of polar and nonpolar groups within the aggregates. The low value of *I*_1_/*I*_3_ in aPMA solutions with *α*_N_ = 0 (*I*_1_/*I*_3_ ≈ 0.9) shows that the core of the aPMA associates is rather hydrophobic (even more than the environment sensed by pyrene in toluene or benzene, where the *I*_1_/*I*_3_ ratio is around 1.1 [53]). Such low *I*_1_/*I*_3_ values may only come from the hydrophobic CH_3_ groups of aPMA that hide inside the core, whereas the polar carboxyl groups reside in the corona and grant the solubility of aPMA in water at *α*_N_ = 0. The basis for such a distribution is the irregular stereo-structure and high flexibility of the atactic chain, which both allow effective hiding of the CH_3_ groups inside the aggregate.

In comparison to aPMA (*α*_N_ = 0), the micropolarity of the iPMA (*α*_N_ ≈ 0.2) aggregates in NaCl and MgCl_2_ is considerably higher as suggested by the *I*_1_/*I*_3_ values around 1.5. It is convenient to compare these values with those for pyrene dissolved in some organic solvents containing C=O or OH groups (the same as in COOH). For example, *I*_1_/*I*_3_ is 1.3 in methanol, 1.35 in ethyl acetate and 1.6–1.8 in water [54]. The C=O and OH groups (not the CH_3_) are obviously the ones that determine the micropolarity of the interior of iPMA aggregates.

Taking into account the high association tendency and high internal polarity of the iPMA aggregates, we propose the following explanation of the above results. The ionized (COO^−^) and unionized carboxyl (COOH) groups within the iPMA aggregates are segregated. The COO^−^ groups are in contact with water (and ensure the solubility of the aggregates), whereas COOH ones constitute the core and can form very strong and cooperative intermolecular H-bonds due to their suitable isotactic orientation on the chain. We propose that the isotacticity enables a very effective “zipper-like” mechanism of inter-chain connections via H-bonds. Although a common notion is that ionization of groups in weak polyelectrolytes takes place statistically along the chain (i.e., that charged and protonated groups are evenly distributed, giving the polyion some average (effective) charge density), there is actually a tendency for the concentration of charges to always be larger at chain ends. This trend may even be larger with the isotactic chains. The existence of the iPMA aggregates at *α*_N_ = 0.2 can only be imagined by proposing the segregation of COO^−^ and COOH groups.

Thus, with iPMA the ionized carboxyl groups (together with the hydrophobic CH_3_ groups) mostly form the outer, water swollen layers of the microgel-like aggregates, whereas the unionized COOH groups reside in the core and contribute to strong and highly cooperative H-bonded structure. This leads to a more contracted core in comparison to aPMA. Such chemical composition of the iPMA aggregate interior results in *I*_1_/*I*_3_ values that are comparable to methanol and ethyl acetate. If too few carboxyl groups are ionized, such an associate/aggregate is not soluble in water. The highly cooperative H-bonding ability between iPMA chains was also proven by molecular modeling [48,55].

In case of iPMA solution with added La^3+^ cations, the *I*_1_/*I*_3_ (≈1.1) is distinctively lower in comparison with NaCl and MgCl_2_ solutions; it is closer to values obtained in aPMA solutions. Along with this, the size of associates of both iPMA and aPMA is also similar in LaCl_3_ solutions. It is *R*_h,ass_ = 200 nm (for aPMA at higher *I*) and *R*_h,ass_ = 191 and 183 nm for iPMA and aPMA, respectively, at lower *I* (c.f. Table 3). It was often observed that polyelectrolyte solutions in the presence of La^3+^ ions become rather dense, even gel-like, pointing to some more extensive network formation in solutions, which may be primarily induced by the presence of trivalent La^3+^ and less by the nature of the polymer.

The fluorimetric results enable additional comments on pH measurements. The added Na^+^, Mg^2+^ and La^3+^ ions can exchange H^+^ from COOH groups on aPMA without a problem, because they are mostly placed on the outside of the aggregate and thus are easily accessible. This, together with *α*_N_ close to 0, leads to a strong increase in *α*_i_ (see Table S3 and Figure 2). However, the increase of *α*_i_ is the least noticeable when Mg^2+^ cations are added; it is actually smaller than in the case of Na^+^ ions, although Mg^2+^ is divalent and expected to replace two H^+^ from PMA. We attribute this observation to the fact that the Mg^2+^ cation is strongly hydrated and interacts with COOH groups via water molecules in its hydration sphere. It is firmly established [8,9,13] that Mg^2+^ cations bind to carboxyl groups in so called monodentate fashion (via one oxygen only; see Scheme S1 in SM), keeping most of the water molecules in its coordination sphere, and thus do not replace many H^+^ from PMA. The high value of the shape parameter *ρ* (>0.90; Table 3) for PMA-Mg^2+^ aggregates speak in favor of this assumption and indicates that a lot of solvent (water) is located inside the aggregates (irrespective of chain tacticity) due to this specific type of interaction. On the other hand, the binding of La^3+^ to COOH groups on PMA chains leads to the exchange of 3H^+^ ions from COOH groups, the number of free H^+^ ions in the surrounding solution increases considerably and along with this *α*_i_. Clearly, the La^3+^ binding is strongly entropically favored [7]. In case of Mg^2+^, the driving force for its binding to COOH groups is weaker. Its extensive hydration prevents “direct” binding of Mg^2+^ ions to the negatively charged carboxylates. The increase in entropy of the system due to the approximately 1:1 exchange of H^+^ by Mg^2+^ is negligible.

In iPMA associates, the unionized COOH groups are mostly hidden inside the aggregates, as proposed above, and thus less accessible for exchange with the added counterions. The interaction of the metal ions with the aggregate surface proceeds via ionized COO^−^ groups and is predominately electrostatic in nature. This may be an additional reason why the added salts, irrespective of cation charge, have a negligible effect on the ionization degree of iPMA with a relatively high average *α*_N_ value and an even higher local (surface) *α*_N_. No changes in pH (*α*_i_) could be observed even in the presence of the highest valency La^3+^ ions. Clearly, electrostatics also conceals any more specific effects of the cations.

With this background knowledge on the state of PMA chains in aqueous solutions at low *α*_N_, we now turn to the calorimetry results. As can be seen from Figure 1, the Δ*H*_ion_ versus *α*_N_ curves in aPMA and iPMA solutions depend strongly on the valency of the added cation. This is most obvious with trivalent La^3+^ ions, where the Δ*H*_ion_ curve for iPMA clearly displays two peaks (Figure 1d), a higher one at low *α*_N_ (<0.25) and a broader one at higher *α*_N_ (peak at *α*_N_ ≈ 0.3). Previously, a precise analysis of the Δ*H*_ion_ = *f*(*α*_N_) curve in NaCl solution [26] also revealed that the broad peak obtained in that case is a result of two overlapping peaks. The same is assumed for the LaCl_3_ solutions in here, and also for the MgCl_2_ ones, although the two peaks are not resolved in the presence of MgCl_2_, but are probably merged together. Clearly, the de-association and change in conformation either take place consecutively (the case in NaCl and very clearly in LaCl_3_) or simultaneously (in MgCl_2_). The shift of the peaks in Δ*H*_ion_ = *f*(*α*_N_) curve in LaCl_3_ (or MgCl_2_) solution to higher *α*_N_ in comparison with NaCl solution may be due to (i) higher charge of La^3+^ (Mg^2+^) ions, which, through screening of electrostatic repulsions between polyions, shift the change in chain conformation to higher *α*_N_ and thus facilitate association, to (ii) different geometry of complexes between these two ions and carboxyl groups and also to (iii) more space demanding interaction if ions are strongly hydrated. These effects are discussed below in more detail.

**Figure 4:**
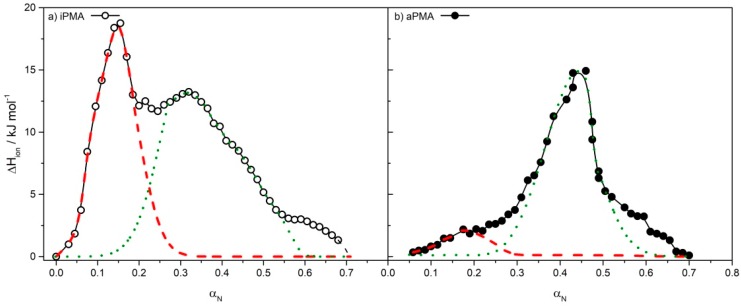
Deconvolution of the calorimetric curves for (**a**) iPMA and (**b**) aPMA in 0.00167 M LaCl_3_ at 25 °C. The lines present two superimposed peaks, obtained by deconvolution of the experimental curve, one in the low (red line) and the other one in the high *α*_N_ region (green line); they represent contributions of the conformational transition (Δ*H*_conf_; green dotted line) and intermolecular association (Δ*H*_ass_; red dashed line) to the total Δ*H*_tr_ value (for details see text).

In order to evaluate separate contributions of Δ*H*_conf_ and Δ*H*_ass_ to Δ*H*_tr_, deconvolution of the calorimetric curves was performed. This could be done reliably for both PMAs for calorimetric curves measured in 0.00167 M LaCl_3_. The deconvolution (see Figure 4) clearly shows that pronounced intermolecular association/aggregation is the main reason why Δ*H*_tr_ values for iPMA are higher than those for aPMA. In the iPMA case, the conformational transition contributes Δ*H*_conf_ = 3.23 kJ·mol^−1^ (*α*_N_ region 0.15–0.60; green dotted line), while association with eventual precipitation (*α*_N_ < 0.30; red dashed line) contributes Δ*H*_ass_ = 2.33 kJ·mol^−1^. The conformational term is thus somewhat larger than the association term (Δ*H*_conf_:Δ*H*_ass_ ≈ 1.4:1). Similar Δ*H*_conf_:Δ*H*_ass_ ratio was obtained previously for iPMA in 0.01 M NaCl at 25 °C; in that case the Δ*H*_conf_ and Δ*H*_ass_ contributions were approximately equal (Δ*H*_conf_:Δ*H*_ass_ ≈ 1:1) [26]. On the contrary, Δ*H*_conf_ (= 2.29 kJ·mol^−1^) for aPMA is considerably larger than Δ*H*_ass_ (= 0.29 kJ·mol^−1^); it contributes almost 90% to the total Δ*H*_tr_ value (Δ*H*_conf_:Δ*H*_ass_ ≈ 8:1), meaning that intermolecular association is far less pronounced than with iPMA, although the aPMA charge is lower. Also, the total Δ*H*_tr_ value for aPMA in 0.00167 M LaCl_3_ is around 2-times smaller in comparison with iPMA. It has to be stressed, however, that the exact nature of the conformational transition in the presence of LaCl_3_ is not clear.

Results in MgCl_2_ solutions deserve a separate discussion. Shapes of calorimetric curves for both PMAs in the presence of Mg^2+^ are similar (see Figure 1c) and distinctively different from the ones obtained in the presence of Na^+^ and La^3+^ ions, as they display a clear plateau for *α*_N_ > 0.55, which is absent in NaCl and LaCl_3_ solutions. We suggest that these differences arise from the monodentate binding of Mg^2+^ ions to carboxylate groups [8,9,13], while La^3+^ ions bind in a bidentate fashion [29,35,36]. Such different behavior arises from special properties of Mg^2+^, which make this cation in comparison with the other two (Na^+^ and La^3+^) very unique. The ionic radius of the Mg^2+^ is smaller (0.65 Å [12]) than the ionic radii of Na^+^ (0.95 Å [12]) and La^3+^ (1.18 Å [22]), thus bare Na^+^ and La^3+^ ions can easily bind to larger and bulkier ligands [8,9,13]. However, the radius of the hydrated form of Mg^2+^ ion (4.76 Å [10,12]) is significantly larger (more than seven times) than its ionic radius (the dehydrated form), while for Na^+^ and La^3+^ ions, their hydration radii are not that much bigger than the ionic ones, approximately 2.75 [12] and 3.10 Å [21], respectively. It is thus not surprising that the exchange rate of water molecules in the hydration layer of Mg^2+^ ions is 10^5^ s^−1^, whereas it is much faster with Na^+^ and La^3+^ ions (around 10^8^ s^−1^) [12,56,57]. This strong interaction of Mg^2+^ ions with water is also the underlying reason for the monodentate type of binding with ligands, which grants more space for the participation of water molecules in the metal complex with the carboxylate [8]. This scenario clearly explains our LS (higher *ρ* values) and pH (no effect on *α*_i_) results. Moreover, water molecules in Mg^2+^-containing complexes are also in a different geometry in comparison to those in the hydration shell around Na^+^ or La^3+^ ions. The conclusion is that binding of the rather large hydrated Mg^2+^ ions to carboxylate groups is expected to be weaker in comparison with the hydrated Na^+^ and La^3+^ ions [6,12]. Strong hydration of Mg^2+^ ions might also be the reason for significantly lower heat effects in aPMA case in comparison with iPMA. Morcellet [33] also reported less endothermic heat effects for the formation of complexes between Mg^2+^ and sPMA, which is known to behave similarly to aPMA. sPMA-Mg^2+^ complexes were also shown to be more stable than iPMA-Mg^2+^ ones, which was attributed to less steric hindrance (or more suitable geometry) in complexation of Mg^2+^ with sPMA.

This is all in agreement with our calorimetric observations (Δ*H*_tr_ for aPMA in the presence of Mg^2+^ is lower than that for iPMA; Table 2) and also with LS results. Due to the monodentate nature of Mg^2+^ binding microgel-like iPMA-Mg^2+^ and aPMA-Mg^2+^ associates contain more water in their interior and are thus highly swollen and also more permeable for the solvent molecules. All this leads to higher *ρ* (≈ 0.9–1) values, i.e., to a more even mass distribution within the particles in comparison to associates of both PMAs with Na^+^ and La^3+^ ions (*ρ* ≈ 0.69–0.77).

The final comment concerns the comparison of aPMA and iPMA aggregate sizes. aPMA forms larger associates in 0.0333 M MgCl_2_ (*R*_h,ass_ = 178 nm) in comparison with iPMA in 0.0033 M MgCl_2_ (*R*_h,ass_ = 75 nm is 2.4-times smaller), although the *M*_w_ values of both PMAs are not that different (*M*_w_ = 159,900 g·mol^−1^ for aPMA and *M*_w_ = 69,500 g·mol^−1^ for iPMA; see Materials and Methods). One of the reasons for this may be the around ten-times higher ionic strength of the medium in the aPMA (*α*_N_ = 0) case. However, at *α*_N_ = 0 the screening effect of salt is supposed to be minor, whereas hydration effects dominate. On the other hand, *R*_h,ass_ values in the presence of La^3+^ cations show no significant differences in size between aPMA and iPMA (*R*_h,ass_ is 180–200 nm in both cases), which might be due to stronger binding of this trivalent cation to carboxylate groups [6,29,36] and very efficient displacement of H^+^ ions from unionized aPMA which is not the case with Mg^2+^ cations.

## 5. Conclusions

The LS and calorimetric measurements in iPMA and aPMA solutions in NaCl, MgCl_2_ and LaCl_3_ show that both PMAs form intermolecular associates at low *α*_N_. Associates in iPMA solutions were studied at a ten-times lower *I* than in aPMA solutions due to precipitation of iPMA from MgCl_2_ and LaCl_3_ salt solutions at high *I* (>0.01). The structure of aggregates is similar to microgel-like particles with values of the shape parameter *ρ* in NaCl and LaCl_3_ solutions varying around 0.70 in the iPMA case and between 0.70 and 0.80 in the aPMA case. However, characteristics of PMAs associates in the presence of Mg^2+^ stand out. The calculated *ρ* values are higher at around 0.90, indicating that associates in MgCl_2_ solutions do not have a core-shell structure, but poses a more homogeneous mass distribution and are permeable for solvent molecules throughout.

Intermolecular association as evidenced by LS has a pronounced effect on calorimetric curves of iPMA and aPMA. It contributes an additional endothermic effect to the measured ionization enthalpies. The size of this heat effect depends on the stereoregularity of the PMA chain and also on the valency of the metal cation present in solution. The calorimetric curves in MgCl_2_ solutions differ from the ones in NaCl and LaCl_3_ solutions due to a different mode of interaction of Mg^2+^ with carboxylate groups. Mg^2+^ ions typically favor monodentate binding to carboxylate groups, while trivalent cations (such as La^3+^) usually bind to COO^−^ in a bidentate fashion, which results in a much stronger binding. This is supported by pH measurements, showing that the La^3+^ ions effectively displace H^+^ from the COOH groups.

Apart from the process of intermolecular association, the conformational transition is also reflected in the calorimetric curves. These two processes may take place either simultaneously (in MgCl_2_) or separately (in NaCl and LaCl_3_). For iPMA in 0.00167 M LaCl_3_, it was possible to determine both contributions through peak deconvolution, which resulted in higher Δ*H*_ass_ values for iPMA in comparison to aPMA. This means that intermolecular association is more pronounced in iPMA (*α*_N_ = 0.22) solutions than in aPMA (*α*_N_ = 0) ones, in spite of a considerably higher charge of the former.

## Figures and Tables

**Table 1:** Final ionic strengths (*I*) and corresponding molar concentrations (*c*_s_) of added salt (NaCl, MgCl_2_, LaCl_3_) solutions for aPMA (*c*_p_ = 0.023 mol·L^−1^, *α*_N_ = 0) and iPMA (*c*_p_ = 0.022 mol·L^−1^; *α*_N_ values are given in the Table) in samples used for LS, pH and fluorimetric measurements.

	iPMA			aPMA	
Added Salt	*I*/mol·L^−1^	*c*_s_/mol·L^−1^	*α*_N_	*I*/mol·L^−1^	*c*_s_/mol·L^−1^
NaCl	0.01	0.01	0.19	0.1	0.1
	0.02	0.02	0.19	0.2	0.2
MgCl_2_	0.01	0.0033	0.19	0.1	0.033
LaCl_3_	0.005	0.00083	0.22	0.05	0.0083
				0.1	0.0167

**Table 2:** Values of the transition enthalpies (Δ*H*_tr_) of iPMA and aPMA (*c*_m_ = 1 g·L^−1^) in aqueous NaCl, MgCl_2_ and LaCl_3_ solutions with *I* = 0.01 mol·L^−1^ at 25 °C.

	iPMA	aPMA
Added Salt	Δ*H*_tr_/kJ·mol^−1^	Δ*H*_tr_/kJ·mol^−1^
0.01 M NaCl	4.83	1.12
0.0033 M MgCl_2_	5.52	0.83
0.00167 M LaCl_3_	5.77	2.97

^1^ Note that Δ*H*_tr_ = Δ*H*_conf_ + Δ*H*_ass_ (see text).

**Table 3:** Hydrodynamic radii (*R*_h,ass_), radii of gyration (*R*_g,ass_) and parameter *ρ* (= *R*_g,ass_/*R*_h,ass_) for associates of iPMA (*α*_N_ ≈ 0.2; see third column) and aPMA (*α*_N_ = 0) in the presence of NaCl, MgCl_2_ and LaCl_3_ at different *I*; *c*_m_ for both PMAs is 2 g·L^−1^ and temperature is 25 °C.

	iPMA					aPMA			
Added Salt	*I*/mol·L^−1^	*α*_N_	*R*_h,ass_/nm	*R*_g,ass_/nm	*ρ*	*I*/mol·L^−1^	*R*_h,ass_/nm	*R*_g,ass_/nm	*ρ*
NaCl	0.01	0.19	59	/	/	0.1	172	126	0.73
	0.02	0.19	80	57	0.71	0.2	177	131	0.74
MgCl_2_	0.01	0.19	75	73	0.97	0.1	178	164	0.92
LaCl_3_	0.005	0.22	191	132	0.69	0.05	183	142	0.77
						0.1	200	152	0.76

**Table 4:** The intensity ratio (*I*_1_/*I*_3_) of vibrational peaks in the fluorescence emission spectrum of pyrene in iPMA (*α*_N_ = 0.19) and aPMA (*α*_N_ = 0) solutions in aqueous NaCl, MgCl_2_ and LaCl_3_ with different ionic strength *I*.

	iPMA		aPMA	
Added Salt	*I*/mol·L^−1^	*I*_1_/*I*_3_	*I*/mol·L^−1^	*I*_1_/*I*_3_
/	/	1.51	/	0.90
NaCl	0.01	1.50	0.1	0.89
	0.02	1.48	0.2	0.90
MgCl_2_	0.01	1.49	0.1	0.89
LaCl_3_	0.005 ^(a)^	1.09	0.05	0.98
			0.1	0.94

^(a)^*α*_N_ = 0.22.

## Supplementary Materials

The following are available online at https://www.mdpi.com/2073-4360/11/4/605/s1, Scheme S1: Schematic presentation of monodentate binding of COO^−^ to Mg^2+^, Scheme S2: A typical structure of a micro-gel like particle, Figure S1: The distribution of hydrodynamic radii (*R*_h_) of particles obtained in i- and aPMA solution in the presence of Mg^2+^, Figure S2: Examples of *R*_h,ass_ determination for i- and aPMA associates in MgCl_2_ solution, Figure S3: Examples of the Debye-Bueche plots for *R*_g,ass_ determination for i- and aPMA associates in MgCl_2_ solution, Figure S4: Ionization enthalpies of i- and aPMA in solutions of MgCl_2_ and LaCl_3_ with *I* = 0.1 mol·L^−1^, Table S1: Hydrodynamic radii for smaller particles (*R*_h,1_) and associates (*R*_h,ass_), radii of gyration (R_g,ass_) and parameter *ρ* in iPMA solutions in the presence of NaCl, MgCl_2_ and LaCl_3_ at different *I*, Table S2: Hydrodynamic radii for smaller particles (*R*_h,1_, *R*_h,2_) and associates (*R*_h,ass_), radii of gyration (R_g,ass_) and parameter *ρ* in aPMA solutions in the presence of NaCl, MgCl_2_ and LaCl_3_ at different *I*, Table S3: The measured pH and calculated *α*_i_ values for i- and aPMA in aqueous NaCl, MgCl_2_ and LaCl_3_ with different *I*.
