# Supplementary material for: Effect of Multivalent Cations on Intermolecular Association of Isotactic and Atactic Poly(Methacrylic Acid) Chains in Aqueous Solutions
Source: Polymers (Basel). 2019 Apr 2;11(4):605. doi: 10.3390/polym11040605 (PMC6523823; doi:10.3390/polym11040605)
Supplement: Supplementary file 1 [file polymers-11-00605-s001.pdf]

Supplementary Materials for the paper

Effect of multivalent cations on intermolecular association of isotactic and atactic poly(methacrylic acid) chains in aqueous solutions

Patricija Hriberšek [1] and Ksenija Kogej [1,*]

[1] Department of Chemistry and Biochemistry, Faculty of Chemistry and Chemical Technology, University of Ljubljana, SI-1000 Ljubljana, Slovenia; patricija.hribersek@fkkt.uni-lj.si; ksenija.kogej@fkkt.uni-lj.si

* Correspondence: ksenija.kogej@fkkt.uni-lj.si; Tel.: +386-1-479-8538

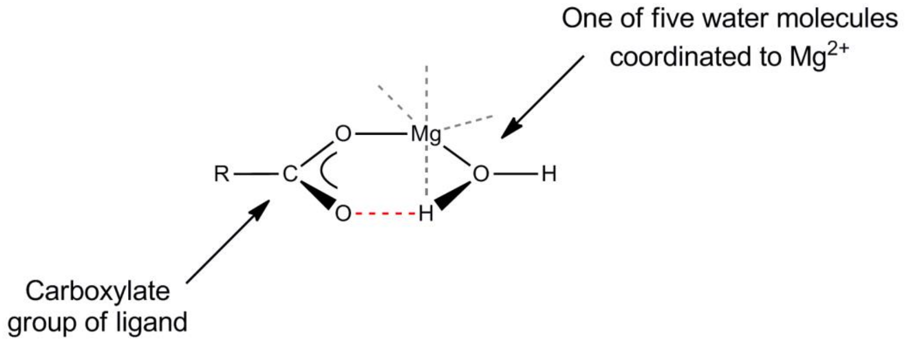

Scheme S1. Schematic presentation of monodentate binding of COO^- to Mg^{2+}. Presented are the COO^- group, one of the five water molecules coordinated to Mg^{2+} (the position of the other four is indicated with grey dashed lines), and favourable hydrogen-bonding (red dashed bond) interaction between this water molecule and metal-free oxygen of COO^-.

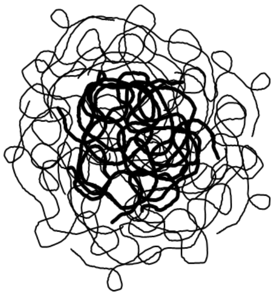

Scheme S2. A typical structure of a microgel-like particle with a compact core, impermeable for the solvent, and a swollen outer corona that allows the solvent to penetrate through. aPMA forms particles with less compact cores, while iPMA associates have denser core due to hydrogen bonding between COOH groups.

Materials and Methods

Light scattering. Methodological aspects of dynamic (DLS) and static (SLS) light scattering can be found elsewhere [1,2,3]. Detailed aspects of data analysis used in this paper are also presented in previous publications [4,5]. Below we shortly give the approach used in our manuscript.

Correlation functions of intensity of scattered light ($G_2(t)$) were recorded at an angle θ simultaneously with the integral time averaged intensities ($I_\theta \equiv I_q$, where $q = (4\pi n_o/\lambda_o)\sin(\theta/2)$ is the scattering vector, n_o is the refractive index of the medium and λ_o is the wavelength of the incident light). Intensities measured in counts of photons per second (cps) were normalized with respect to the Rayleigh ratio of toluene thus converting the cps-units into absolute intensity units given in cm^{-1}. In order to determine the hydrodynamic radii of particles, $G_2(t)$ was converted into the correlation function of the scattered electric field ($g_1(t)$) by using the Siegert's relationship [1,2]:

$$g_2(t) = 1 + \beta|g_1(t)|^2$$

or

$$|g_1(t)| = \beta^{-\frac{1}{2}}\sqrt{\frac{G_2(t)}{G_2(\infty)} - 1}$$

where $G_2(\infty)$ is the experimentally determined baseline, $g_2(t) = G_2(t)/G_2(\infty)$, and β is the coherence factor determined by the geometry of the detection. Usually it is between $0.5 \leq \beta \leq 0.8$. The $g_1(t)$ function was analyzed by CONTIN analysis in order to determine distributions of the hydrodynamic radii (R_h) of particles in solutions. Example of such distribution obtained at a fixed q is presented in Figure S1 and was used to estimate the mean hydrodynamic radii of larger particles ($R_{h,ass}$; *c.f.* peak 2 in Figure S1a and peak 3 in Figure S1b) and also to split the total intensity of scattered light into contributions of individual populations. This was described in detail in [6].

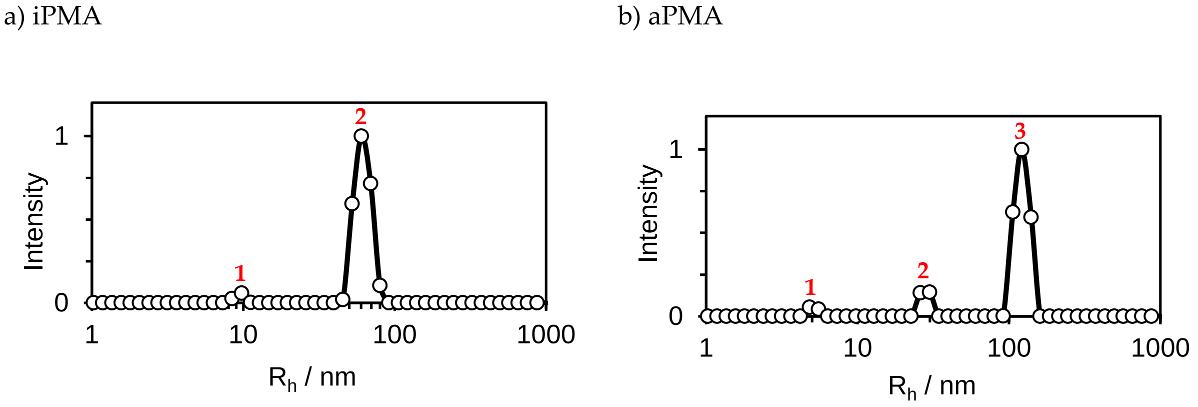

Figure S1. The distribution of hydrodynamic radii (R_h) of particles obtained in solutions of a) iPMA (c_p = 0.022 mol L^{-1}, α_N = 0.19) in 0.0033 M $MgCl_2$ and b) aPMA (c_p = 0.023 mol L^{-1}, α_N = 0) in 0.0333 M $MgCl_2$ at θ = 90°. In the iPMA case, peak 1 corresponds to particles with $R_{h,1}$ and peak 2 to associates with $R_{h,ass}$. In the aPMA case, peaks 1 and 2 correspond to individual chains and possibly smaller associates with $R_{h,1}$ and $R_{h,2}$, respectively, while peak 3 applies to larger aggregates with $R_{h,ass}$.

Hydrodynamic radii of associates ($R_{h,ass}$), which are reported in Tables S1 and S2, were obtained by extrapolation of the $R_{h,app}$ values to $\theta = 0°$ as demonstrated in Figure S2.

R_h of smaller particles and individual chains ($R_{h,1}$ and $R_{h,2}$) reported in Tables S1 and S2 were calculated as the average of R_h values measured at several q values, because they were independent of the angle.

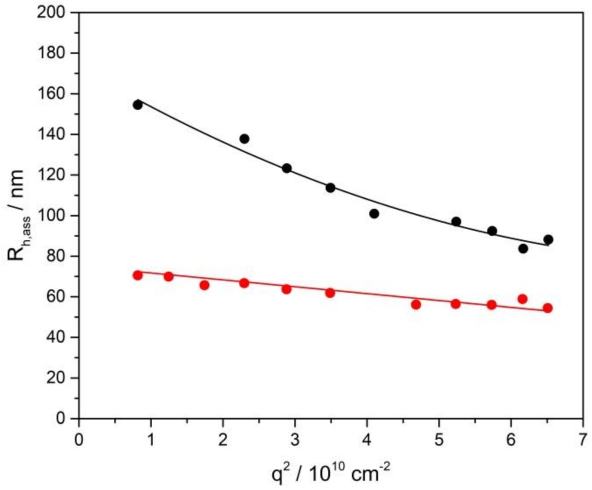

Figure S2. Examples of $R_{h,ass}$ determination as extrapolation of R_h values to $\theta = 0°$ for iPMA (red) associates (c_P = 0.022 mol L^{-1}, α_N = 0.19) in 0.0033 M $MgCl_2$ and for aPMA (black) associates (c_P = 0.023 mol L^{-1}, α_N = 0) in 0.0333 M $MgCl_2$.

From the scattering intensities of the aggregates, the form factor $P(\theta)$ was calculated as $P(\theta) = \frac{I_\theta}{I_0}$. $P(\theta)$ depends on the size and shape of particles and enables the determination of the radius of gyration (R_g) [1,2,3].

The Debye-Bueche scattering function [1,2,3] was found to be the most suitable to determine the radius of gyration of larger particles or associates ($R_{g,ass}$) (see an example of the suitable plot in Figure S3):

$$P(\theta) = \frac{1}{(1 + \frac{(qR_g)^2}{6})^2}.$$

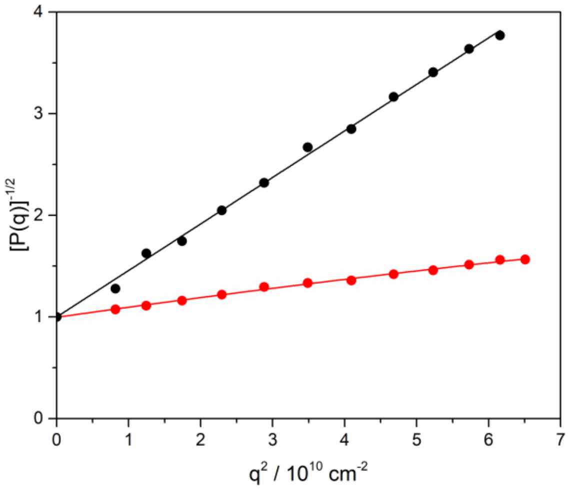

Figure S3. Examples of the Debye-Bueche plots ($1/\sqrt{\mathbf{P(\theta)}}$ vs. q^2) for $R_{g,ass}$ determination for iPMA associates (red points; c_P = 0.022 mol L^{-1}, α_N = 0.19) in 0.0033 M $MgCl_2$ and for aPMA (black) associates (black points; c_P = 0.023 mol L^{-1}, α_N = 0) in 0.0333 M $MgCl_2$.

Results

Calorimetry. As can be seen from Figure S4 ($\Delta H_{ion} = f(\alpha_N)$) for aPMA in 0.0333 M $MgCl_2$ heat effects are rather large (from around 10 to around 30 kJ mol^{-1}) while for aPMA in 0.0167 M $LaCl_3$ they are even higher (up to around 40 kJ mol^{-1}). These values are considerably higher than those for solutions of both PMAs with a ten-times lower I (*c.f.* Figure 1 in the main paper). Polyacids eventually precipitated from these solutions. iPMA in 0.0167 M $LaCl_3$ precipitated after a few addition of the titrant (NaOH) and consequently only a few initial ΔH_{ion} values could be measured at this I.

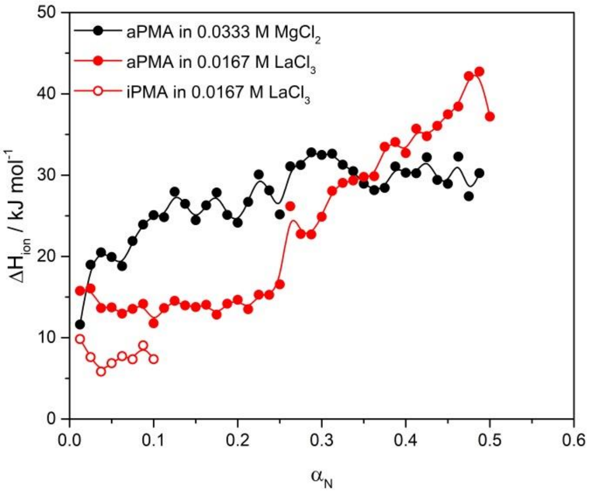

Figure S4. Heat effects (ΔH_{ion} versus α_N curves) measured with ITC during ionization of iPMA and aPMA (c_P = 0.01 mol L^{-1}) in solutions of $MgCl_2$ and $LaCl_3$ with I = 0.1 mol L^{-1}: aPMA in 0.0333 M $MgCl_2$ and in 0.0167 M $LaCl_3$, and iPMA in 0.0167 M $LaCl_3$.

Light scattering.

Table S1. Hydrodynamic radii for smaller particles ($R_{h,1}$) and associates ($R_{h,ass}$), radii of gyration ($\mathrm{R}_{g,ass}$) and parameter ρ in iPMA solutions (c_P = 0.022 mol L^{-1}, different α_N) in the presence of NaCl, $MgCl_2$ and $LaCl_3$ at different I at 25 °C.

**Added salt**	***I* / mol L^{-1}**	α_N	**$R_{h,1}$ / nm**	**$R_{h,ass}$ / nm**	**$R_{g,ass}$ / nm**	ρ
NaCl	0.01	0.19	8	59	/	/
	0.02	0.19	10	80	57	0.71
$MgCl_2$	0.01	0.19	10	75	73	0.97
$LaCl_3$	0.005	0.22	16	191	132	0.69

Table S2. Hydrodynamic radii for smaller particles ($R_{h,1}$, $R_{h,2}$) and associates ($R_{h,ass}$), radii of gyration ($\mathrm{R}_{g,ass}$) and parameter ϱ in aPMA solutions (c_P = 0.023 mol L^{-1}, α_N = 0) in the presence of NaCl, $MgCl_2$ and $LaCl_3$ at different I at 25 °C.

**Added salt**	***I* / mol L^{-1}**	**$R_{h,1}$/ nm**	**$R_{h,2}$ / nm**	**$R_{h,ass}$ / nm**	**$R_{g,ass}$ / nm**	ρ
NaCl	0.1	/	15	172	126	0.73
	0.2	/	13	177	131	0.74
$MgCl_2$	0.1	6	18	178	164	0.92
$LaCl_3$	0.05	6	12	183	142	0.77
	0.1	9	15	200	152	0.76

pH measurements.

Table S3. The measured pH and calculated α_i values for iPMA (different α_N) and aPMA (α_N = 0) in aqueous NaCl, $MgCl_2$ and $LaCl_3$ with different I.

iPMA					aPMA			
**Added salt**	**I / mol L^{-1}**	α_N	**pH**	α_i	**Added salt**	**I / mol L^{-1}**	**pH**	α_i
/	/	0.19	5.820	0.190	/	/	3.56	0.012
NaCl	0.01	0.19	5.840	0.190	NaCl	0.1	3.31	0.021
	0.02	0.19	5.770	0.190		0.2	3.28	0.023
						0.3	3.24	0.025
						0.4	3.2	0.027
						0.5	3.17	0.029
$MgCl_2$	0.01	0.19	5.680	0.190	$MgCl_2$	0.1	3.52	0.013
						0.2	3.45	0.015
						0.3	3.42	0.016
						0.4	3.42	0.016
						0.5	3.44	0.016
/	/	0.22	5.980	0.220	$LaCl_3$	0.05	3.14	0.031
$LaCl_3$	0.005	0.22	5.560	0.220		0.1	3.02	0.041
						0.2	2.91	0.053
						0.3	2.87	0.058
						0.4	2.82	0.066
						0.5	2.77	0.072

References

[1] Schärtl, W. *Light Scattering from Polymer Solutions and Nanoparticle Dispersions*; Springer Verlag: Berlin, Heidelberg, 2007.

[2] Brown, W. *Dynamic Light Scattering: The Method and Some Application*; Clarendon Press: Oxford, 1993.

[3] Kratochvil, P. Particle scattering functions. In *Particle scattering functions in light scattering from polymer solutions;* Huglin M.B. Ed.; Academic Press Inc.: London and New York, 1972; pp. 333–384.

[4] Sitar, S.; Aseyev, V.; Kogej, K. Differences in association behaviour of isotactic and atactic poly(methacrylic acid). *Polymer* **2014**, *55*, 848–854.

[5] Sitar, S.; Aseyev, V.; Kogej, K. Microgel-like aggregates of isotactic and atactic poly(methacrylic acid) chains in aqueous alkali chloride solutions as evidenced by light scattering. *Soft Matter* **2014**, *10*, 7712–7722.

[6] Tarassova, E.V.; Aseyev, V.; Filippov, A.; Tenhu, H. Structure of poly(vinyl pyrrolidone) – C_{70} complexes in aqueous solutions. *Polymer* **2007**, *48*, 4503-4510.
